# Effects of SIRT7 Inhibition on Milk Fat Synthesis and Fatty Acid Composition in Dairy Goat Mammary Epithelial Cells

**DOI:** 10.3390/ani16182886

**Published:** 2026-09-14

**Authors:** Jun Li, Zhongying Li, Menghan Han, Mengfan Zhao, Zhuoying Li, Huibin Shi, Haoyuan Han, Kun Liu, Xing Liu, Kai Quan

**Affiliations:** 1 College of Animal Science and Technology, Henan University of Animal Husbandry and Economy, Zhengzhou 450046, China; lijun.nn@163.com (J.L.); 13290790403@163.com (M.Z.); liukun_139@163.com (K.L.); 2 Henan Key Laboratory of Healthy Breeding and Efficient Reproduction of Livestock and Poultry, Zhengzhou 450046, China; 3 College of Animal Science and Technology, Ningxia University, Yinchuan 750021, China; lzy619804@163.com (Z.L.); 18237016008@163.com (M.H.); lzy13323@163.com (Z.L.); 4 Zhengzhou Agricultural Technology Extension Center, Zhengzhou 450006, China; zzmj2008@sina.com

**Keywords:** dairy goat, Sirt7, inhibitor (ID: 97491), milk fat synthesis, fatty acid composition

## Abstract

Milk fat, a key nutritional component of milk, not only supplies energy and immune protection for offspring but also serves as a critical quality indicator for goat milk. Sirt7, one of the least studied members of the Sirtuins family, has been shown to play a regulatory role in lipid metabolism. This study investigated the regulatory role of Sirt7 in milk fat synthesis and fatty acid composition in dairy goat mammary epithelial cells (GMECs) using a Sirt7 inhibitor (ID: 97491). GMECs were treated with a Sirt7 inhibitor (ID: 97491), and cell viability was assessed via the CCK-8 assay, which confirmed that 15 μmol/L inhibitor had no cytotoxic effects. Functional analyses using RT-qPCR, Western blot, and GC-MS revealed that Sirt7 inhibition upregulated the expression of *SREBP1*, *CD36*, *ACC*, *SCD1*, and *ELOVL6* while downregulating *FOXO1* and *FABP1*. At the protein level, the inhibitor reduced Sirt7, FOXO1. Additionally, Sirt7 inhibition increased intracellular lipid droplet and triglyceride content. GC-MS analysis showed that Sirt7 inhibition decreased C11:0; increased C16:0, C15:0, C20:0, C24:0, C16:1, and C18:1n9c; and decreased C20:5n3 (EPA). Collectively, these findings suggest that pharmacological inhibition of Sirt7 is associated with upregulation of SREBP1, disruption of lipid homeostasis, triglyceride accumulation, and altered fatty acid composition in GMECs. These results provide preliminary evidence for a potential regulatory role of Sirt7 in milk fat synthesis and lay a foundation for future mechanistic studies.

## Simple Summary

Sirt7, a member of the NAD^+^-dependent class III histone deacetylase family (Sirtuins), is involved in the regulation of multiple metabolic processes, including glucose homeostasis and energy balance, and plays a key regulatory role in lipid metabolism. The present study demonstrates that inhibition of Sirt7 increases the expression of SREBP1 and downstream lipogenic genes, thereby enhancing lipid droplet accumulation and triglyceride levels, while also improving the fatty acid profile. These findings help to elucidate the molecular mechanisms by which Sirt7 regulates lipid metabolism and provide a deeper understanding of the regulatory mechanisms underlying milk fat synthesis in mammary epithelial cells of dairy goats.

## 1. Introduction

Milk fat, a naturally occurring lipid mixture in milk, is a core nutritional component of dairy products. Triglycerides (TGs) are its primary constituent, comprising approximately 95% of total milk fat [1,2]. Milk fat synthesis occurs in mammary epithelial cells and involves the integration of nutrient signaling, hormonal regulation, and intracellular signaling networks, as well as the coordination of de novo fatty acid synthesis, exogenous fatty acid uptake, and triglyceride assembly and secretion [3,4,5]. Ultimately, milk fat is secreted into milk as lipid droplets, which are key determinants of milk quality traits, including fatty acid composition, flavor, and texture [6]. Thus, investigating the molecular mechanisms underlying milk fat synthesis will provide a robust theoretical basis and data-driven support for molecular breeding of milk fat traits in dairy goats.

The Sirtuin family is a class of highly conserved NAD^+^-dependent class III histone deacetylases with multiple enzymatic activities, which exert core regulatory roles in key biological processes such as cellular metabolic regulation, proliferation and apoptosis, and DNA repair [7,8]. In mammals, this family contains seven protein members, namely Silent mating type information regulator 2-related enzyme Sirt1 to Sirt7 [9]. Among them, Sirt7 is the only Sirtuin protein that is mainly localized to the RNA polymerase I transcription site in the nucleolus, and it exerts unique regulatory functions in processes including lipid metabolism, cellular signal transduction, and protein synthesis. In particular, its role in regulating the lipid metabolism network has garnered increasing attention in recent years [10,11]. Existing studies have confirmed that the regulatory function of Sirt7 in lipid metabolism is tissue-specific. Li et al. [12] found in a hypertensive mouse model that overexpression of *Sirt7* in renal tubular epithelial cells can significantly alleviate renal lipid peroxidation injury, and maintain lipid metabolic homeostasis by enhancing the Nrf2/GPX4/xCT and HO-1/NQO1 antioxidant signaling pathways. Furthermore, the study by Cho et al. [13] revealed the regulatory role of Sirt7 in adipogenesis. Specifically, Sirt7 mediates hepatic endoplasmic reticulum stress through the Sirt7/autophagy signaling pathway, thereby modulating lipid deposition in the liver. Their study found that in a palmitate-induced hepatocyte adipogenesis model, knockdown of *Sirt7* can significantly attenuate the inhibitory effect of regulatory factors on lipid accumulation, and reverse their downregulatory effect on the expression of adipogenesis-related proteins. This indicates that Sirt7 can directly participate in the molecular regulation of adipogenesis by regulating the endoplasmic reticulum stress pathway.

Current research on the molecular mechanisms by which Sirt7 regulates lipid metabolism and adipogenesis has predominantly focused on human and mouse models. However, studies investigating the functional role of Sirt7 in ruminants remain relatively limited, primarily concentrating on inflammatory regulation and genetic polymorphisms. It has been reported that Sirt7 alleviates lipopolysaccharide (LPS)-induced inflammatory injury and apoptosis by suppressing the NF-κB signaling pathway [14]. Additionally, Xu et al. [15] showed that Sirt7 Indels are linked to sheep growth traits, indicating their utility as breeding markers. Nevertheless, the specific functions, molecular regulatory pathways, and underlying mechanisms of Sirt7 in ruminant lipid metabolism and adipogenesis remain largely unexplored and warrant further investigation.

Inhibitor (ID: 97491) is a specific small-molecule inhibitor of Sirt7. Studies have shown that it reduces the deacetylase activity of Sirt7 in a dose-dependent manner, with a half-maximal inhibitory concentration (IC50) of 325 nM [16]. Previous studies have shown that SIRT7 facilitates adipogenesis through the inhibition of SIRT1 autocatalytic activation, which reduces SIRT1-mediated p53 deacetylation and establishes a lipogenic cellular milieu; in parallel, compound (ID: 97491), a specific SIRT7 inhibitor, elevates p53 acetylation at lysine residues 373 and 382 (K373 and K382), thereby stabilizing p53 and promoting apoptosis via the caspase pathway—both effects being mechanistically linked to the regulatory network of SIRT7 in lipid metabolism [16,17].

In this study, dairy goat mammary epithelial cells (GMECs) were treated with a Sirt7 inhibitor (ID: 97491), and a *Sirt7* inhibition model was constructed to observe the effects of *Sirt7* suppression on the expression of milk fat metabolism-related genes and proteins, as well as on TG and lipid droplet accumulation. Furthermore, gas chromatography–mass spectrometry (GC-MS) was used to detect the effect of *Sirt7* inhibition on the composition of medium- and long-chain fatty acids. This study aimed to elucidate how the Sirt7 inhibitor (ID: 97491) regulates Sirt7 enzyme activity and downstream signaling networks through multiple mechanisms to reveal the role of Sirt7 in milk fat synthesis in GMECs, and to provide a theoretical basis for elucidating its molecular regulatory mechanism.

## 2. Materials and Methods

### 2.1. Ethics Statement

The dairy goat mammary epithelial cells used in this study were previously cryopreserved by the Laboratory of Animal Molecular Genetics, Henan University of Animal Husbandry and Economy (Zhengzhou, China). Their purification and identification were performed following the method described by Wang et al. [18]. The animal procedures described in this study were examined and approved by the Ethics Committee of Henan University of Animal Husbandry and Economy (Approval No. HNUAHE ER D202302121).

### 2.2. Culture of Dairy Goat Mammary Epithelial Cells

The cryopreserved GMECs at passage 8 were thawed and then centrifuged at 1000 rpm for 5 min. The cells were resuspended in complete medium consisting of 90% DMEM/F 12 (SH30023.01, HyClone, Logan, UT, USA), 10% FBS (081-150, Wisent, Saint-Bruno-de-Montarville, QC, Canada), 100 U/mL penicillin–streptomycin (080092569, Harbin Pharmaceutical Group, Harbin, China), 5 μg/mL hydrocortisone, 5 μg/mL insulin (P3376, Beyotime, Shanghai, China), and 10 ng/mL epidermal growth factor. Cells were cultured at 37 °C in a 5% CO_2_ incubator (Thermo Fisher Scientific, 50163009, Beijing, China). Subculture was performed when cell density reached more than 90% confluence under an inverted microscope (Nikon Corporation, ECLIPSE Ts2, Tokyo, Japan).

### 2.3. Cell-Counting Kit-8 (CCK-8) Assay

GMECs were seeded into 96-well cell culture plates (Corning Incorporated, Corning, NY, USA) at a density of 5 × 10^3^ cells per well. When cell confluence reached 70–80%, the cells were treated with gradient concentrations of the Sirt7 inhibitor (ID: 97491) (0, 5, 10, 15, and 20 μmol/L) according to the dosage used by Kim et al. [16] Each concentration was applied in six replicates. After 48 h of treatment, the culture medium was discarded, and 10% CCK-8 reagent (C0041, Beyotime, Shanghai, China) was added to each well. Following incubation at 37 °C in a 5% CO_2_ atmosphere for 2 h, the absorbance at 450 nm was measured using a microplate reader (Thermo Fisher Scientific, Multiskan FC, Beijing, China). The concentration with the minimal inhibitory effect on GMEC viability was selected for subsequent experiments.

### 2.4. Total RNA Isolation and Reverse Transcription Quantitative PCR Analysis

Following 48 h of cellular treatment, total RNA was isolated from GMECs with TRIpure reagent (RN0101, Aidlab, Beijing, China) in strict accordance with the manufacturer’s operational protocols. The ubiquitously expressed transcript (*UXT*, *RPS9*, *MRPL39*) gene was selected as the internal reference gene for the normalization of relative gene expression levels. Gene-specific primers for the goat *Sirt7* gene (NCBI accession No. XM_018065444.1) were designed based on its conserved region using Primer Premier 5.0 software. The primer sequences for all target genes are provided in Table 1. Qualified total RNA was then reverse-transcribed using the Tiangen reverse transcription kit (KR128-01, Beijing, China). The expression levels of relevant genes were detected using the Tiangen SYBR Green fluorescence quantitative kit (FP205-01, TIANGEN, Beijing, China). The reaction system consisted of 2× SuperReal PreMix Plus (10 μL), 50× ROX Reference Dye (0.4 μL), forward primers (0.6 μL), reverse primers (0.6 μL), cDNA (1 μL), and ddH_2_O (7.4 μL), for a total volume of 20 μL. All qPCR amplification reactions were carried out on a QuantStudio™ 6 Pro Real-Time PCR System (A43159, Thermo Fisher Scientific, Waltham, MA, USA) with a standard amplification procedure. The thermal cycling protocol consisted of an initial pre-denaturation step at 95 °C for 10 min, followed by 40 repeated cycles of 95 °C for 10 s (denaturation) and 60 °C for 30 s (annealing and extension). Three biological replicates and three technical replicates were established for each sample group to ensure experimental reproducibility and accuracy. The experimental data were analyzed using the 2^−ΔΔCt^ method.

### 2.5. Protein Extraction and Western Blot Analysis

Cells were seeded into 6-well plates (Corning Incorporated, Corning, NY, USA) at a density of 2.5 × 10^5^ cells per well. Following a 48 h treatment with the inhibitor, the cells were collected, and total protein was extracted using RIPA lysis buffer (strong) supplemented with a protease inhibitor cocktail (R0010, Solarbio, Beijing, China). Protein concentrations were measured with a BCA kit (P0010, Beyotime, Shanghai, China). Protein lysates were then mixed with 5 × SDS loading buffer (B1007, Applygen, Beijing, China) at a 1:4 ratio, heated at 95 °C for 10 min, and stored at −20 °C. Denatured proteins were resolved by 12% SDS-PAGE using a gel preparation kit (AP1002, Applygen, Beijing, China), with three replicate wells per group, and subsequently electrotransferred onto polyvinylidene fluoride (PVDF) membranes. After transfer, membranes were blocked with QuickBlock Western blocking buffer (P0240, Beyotime, Shanghai, China) at room temperature. Following removal of the blocking buffer, membranes were incubated overnight at 4 °C with primary antibodies against β-actin (1:2000, DKM041F, Dakome, Beijing, China), Sirt7 (1:1000, bs-5973R, Bioss, Beijing, China), FOXO1 (1:2000, 18592-1-AP, Proteintech, Wuhan, China), and *p*-FOXO1 (1:1000, #9461, Cell Signaling Technology, Danvers, MA, USA). After 12–18 h of incubation, membranes were washed three times with TBST and then incubated for 1 h with goat anti-mouse IgG-HRP (1:3000, DKM030205, Dakome, Beijing, China) and goat anti-rabbit IgG-HRP (1:3000, DKM030212, Dakome, Beijing, China) as secondary antibodies. Protein bands were visualized using an ECL chemiluminescence reagent (P1050, Applygen, Beijing, China) and imaged with a 5200 fully automated chemiluminescence imaging system (Tanon, Shanghai, China).

### 2.6. Triglyceride Determination and Oil Red O Staining

Intracellular TG content was determined using a TG assay kit (A110-1-1, Nanjing Jiancheng Bioengineering Institute, Nanjing, China) following the manufacturer’s instructions. Briefly, treated GMECs were collected and resuspended in 0.2–0.3 mL lysis buffer containing 1–2% Triton X-100, and then incubated for 40 min. Distilled water, calibrator solution, and 2.5 μL of cell lysate were added to blank, standard, and sample wells of a 96-well plate, respectively. All wells were supplemented with 250 μL of working solution and incubated at 37 °C for 10 min. The absorbance was measured at 500 nm using a Multiskan FC microplate reader (Thermo Fisher Scientific, Beijing, China). TG content was calculated within the linear range of the standard curve and normalized to the total protein concentration of cell homogenates.

$$\mathrm{Triglyceride}\;\mathrm{content}\;\left(\mathrm{mmol}/\mathrm{gprot}\right)=\frac{A_{\mathrm{sample}}-A_{\mathrm{blank}}}{A_{\mathrm{standard}}-A_{\mathrm{blank}}}\times C_{\mathrm{standard}}/C_{\mathrm{prot}}$$

C standard: standard concentration, 2.26 mmol/L.

Cpr: homogenate protein concentration of the sample to be tested, gprot/L.

Following 48 h of inhibitor treatment, cellular lipid droplet content was assessed using Oil Red O staining (C0157S, Beyotime, Shanghai, China). Cells were washed twice with PBS (HyClone, Logan, USA), fixed with an appropriate volume of 4% paraformaldehyde (Catalog No. P0099, Beyotime, Shanghai, China) solution at room temperature for 10 min, and then rinsed three times with PBS (SH30256.01, HyClone, Logan, UT, USA). The cells were then immersed in staining wash solution for 20 s, after which the solution was removed, and the cells were stained with Oil Red O solution (C0158, Beyotime, Shanghai, China) for 40 min to label intracellular lipid droplets. Subsequently, the staining solution was discarded, and the cells were washed twice with PBS (HyClone, Logan, USA). After washing, 500 μL of PBS was added to each well, and lipid droplet distribution was observed and photographed using an inverted microscope (Nikon Corporation, ECLIPSE Ts2, Tokyo, Japan).

### 2.7. Measurement of Fatty Acid in GMEC

The cell pellet was collected into a centrifuge tube, and 4 mL of chloroform (Aladdin™ C109595, Aladdin, Shanghai, China) was added, followed by vortex mixing for 30 s. Subsequently, 0.9% NaCl solution (ST341, Beyotime, Shanghai, China) was added and vortexed for 30 s. The mixture was then centrifuged at 3500 rpm for 15 min at room temperature, and the lower organic phase was carefully transferred to a new tube using a pipette. Then, 2 mL of dichloromethane was added, vortexed for 30 s, centrifuged for 15 min, and the bottom layer was removed. This extraction step was repeated twice. The combined lower layers were pooled and dried under a stream of nitrogen gas. After nitrogen drying, 2 mL of methanol (AR, Fuyu, Tianjin, China) (containing 5% sulfuric acid) was added, vortexed for 30 s, and incubated in a water bath at 80 °C for 2 h. After cooling, 2 mL of n-hexane and 1 mL of distilled water were added, vortexed for 30 s, and centrifuged at 2000 rpm for 5 min. The supernatant was collected, mixed with 1 mL of distilled water, vortexed for 30 s, and centrifuged again at 2000 rpm for 5 min. The supernatant was then transferred and evaporated to dryness under a stream of nitrogen gas. Finally, an appropriate volume of isooctane was added according to the sample concentration, vortexed for 30 s, allowed to stand for 5 min, and the resulting solution was transferred to a sample vial for detection. A CPSil 88 column (100 m × 0.25 mm × 0.25 µm, Agilent, Santa Clara, CA, USA) was used. The GC-MS data acquisition system primarily consisted of a gas chromatograph (Agilent 7820, Santa Clara, CA, USA) and a quadrupole mass spectrometer (Agilent 5977, Santa Clara, CA, USA).

### 2.8. Statistical Analysis

All experimental data were analyzed using SPSS 26.0 software (IBM, Armonk, NY, USA) and GraphPad Prism (version 10.0, GraphPad Software, San Diego, CA, USA), and all results are presented as mean ± SEM from three independent replicates. The independent variable was the concentration of the Sirt7 inhibitor (ID: 97491) (0 vs. 15 μmol/L), with 0 μmol/L serving as the control. The dependent variables included Sirt7 expression, lipid metabolism-related gene expression, triglyceride content, lipid droplet accumulation, and fatty acid composition. The statistical significance of differences between the control and inhibitor-treated groups was evaluated by independent unpaired Student’s *t*-test, following confirmation of normality and homogeneity of variances (Shapiro–Wilk and Levene’s tests, both *p* > 0.05). Statistical significance was determined at *p* < 0.05, with “*”: *p* < 0.05 (significant difference) and “**”: *p* < 0.01 (very significant difference).

## 3. Results

### 3.1. Concentration Screening and Potency Testing of the Sirt7 Inhibitor (ID: 97491)

**Figure 1:**
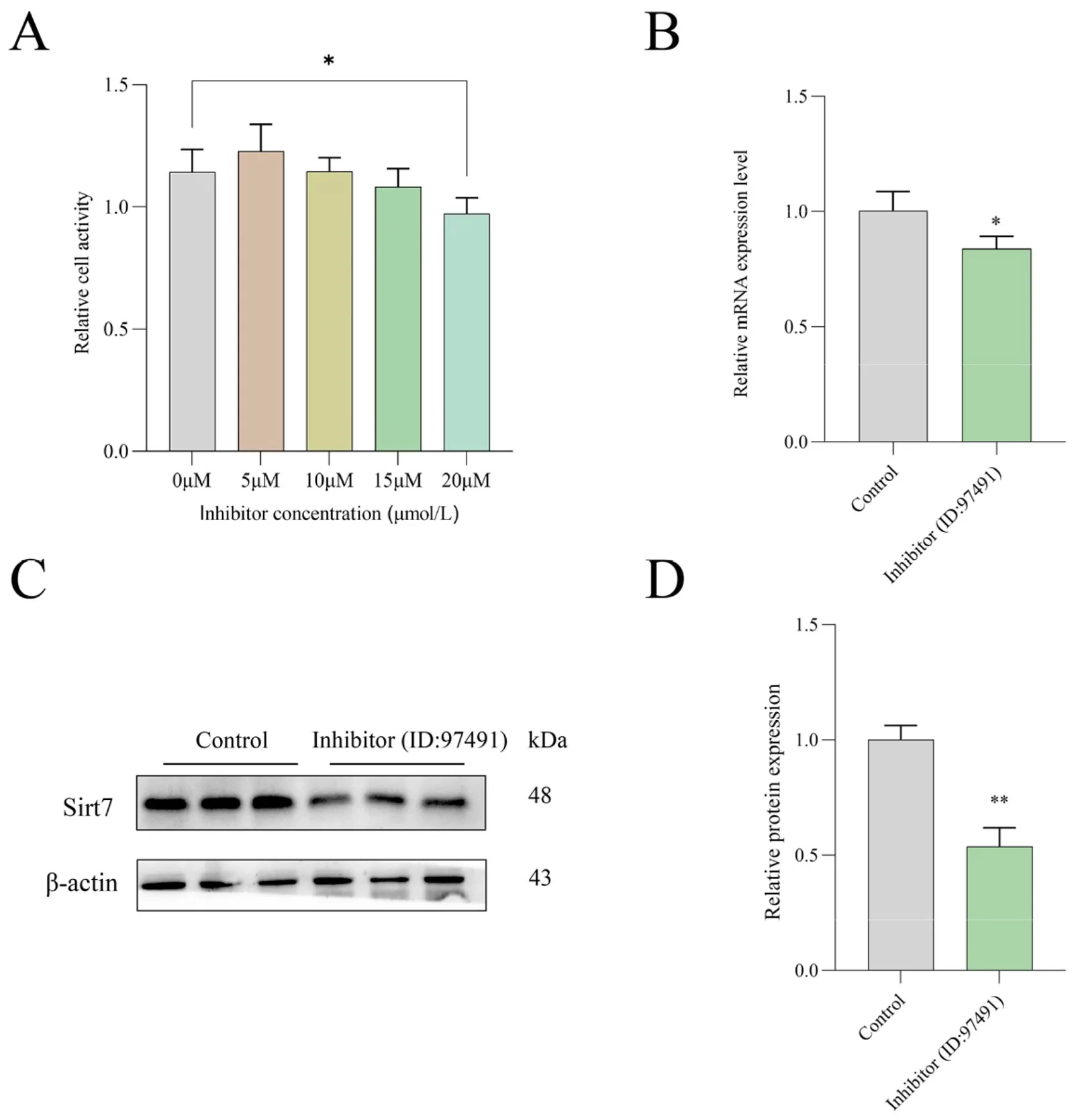
Concentration Screening of the Sirt7 Inhibitor (ID: 97491) and assessment of Sirt7 activity. (**A**) Cell viability was detected using a CCK-8 assay kit following treatment with inhibitor 97491. (**B**) Assessment of *Sirt7* mRNA suppression efficiency using RT-qPCR. (**C**,**D**) Assessment of Sirt7 protein inhibition efficiency using Western blot. * *p* < 0.05; ** *p* < 0.01.

To evaluate the cytotoxicity of the Sirt7 inhibitor (ID: 97491) on GMECs and its inhibitory efficacy against *Sirt7*, the CCK-8 assay was used to assess the effect of the Sirt7 inhibitor (ID: 97491) at concentrations ranging from 0 to 20 μmol/L on cell viability. The 0 μmol/L was used as the control group for intergroup statistical comparisons. The results showed that, compared with the control group, cell viability in the 5, 10, and 15 μmol/L treatment groups exhibited no significant differences (*p* > 0.05). In contrast, cell viability in the 20 μmol/L treatment group was significantly lower than that in the control group (*p* < 0.05, Figure 1A). Therefore, a concentration of 15 μmol/L of the Sirt7 inhibitor (ID: 97491), which had no significant effect on cell viability, was selected for subsequent experiments. At this concentration, *Sirt7* mRNA expression levels decreased by 15–20% (*p* < 0.01, Figure 1B), and Sirt7 protein expression levels decreased by 40–50% (*p* < 0.01, Figure 1C,D).

### 3.2. Effects of Sirt7 Inhibition on Milk Fat Synthesis-Related Genes and Proteins in GMECs

**Figure 2:**
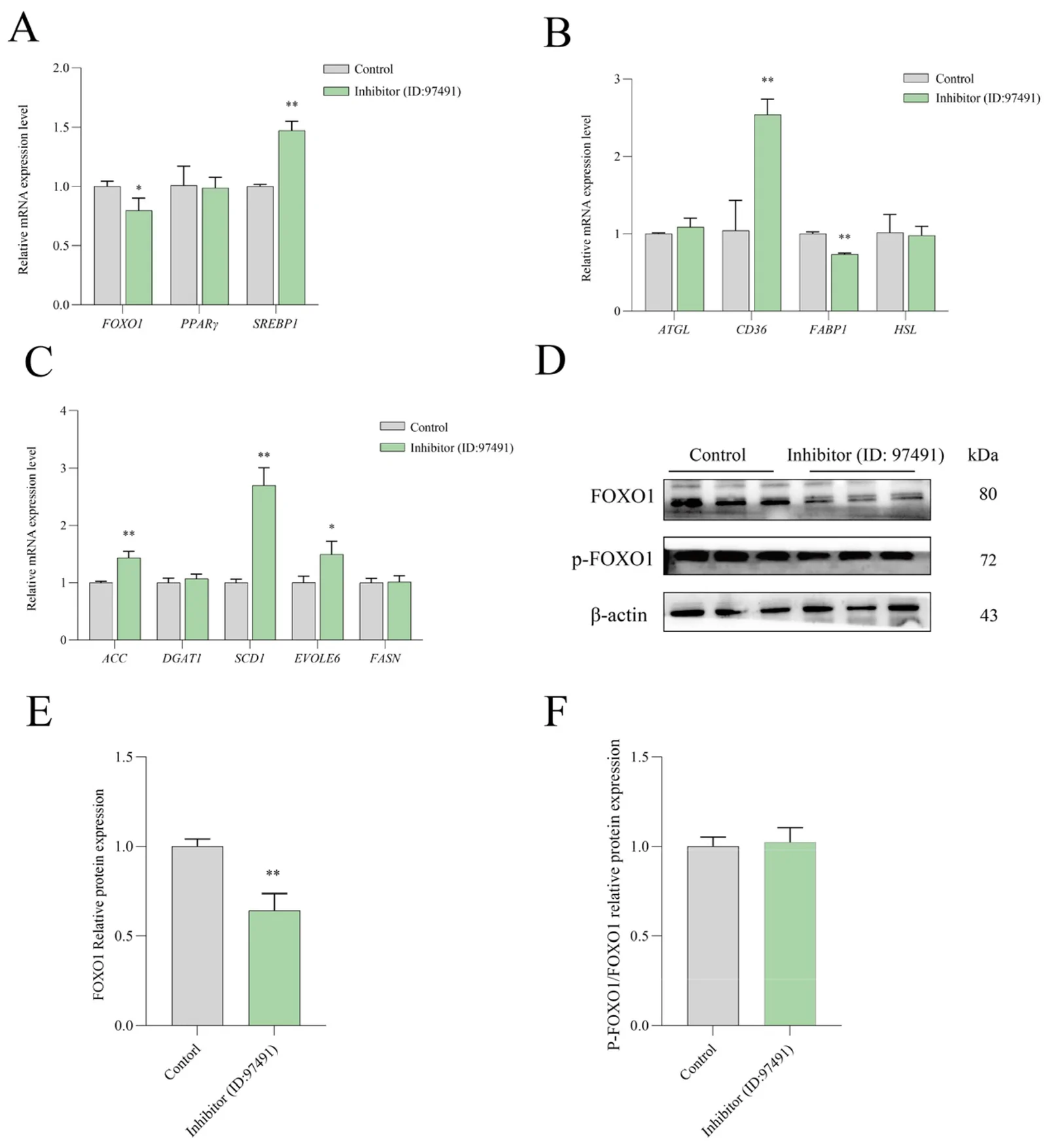
Effects of *Sirt7* inhibition on the expression of lipid metabolism-related genes and proteins in GMECs. (**A**) Expression levels of lipogenic transcription factors; (**B**) expression levels of genes related to lipid transport and lipolysis; (**C**) expression levels of genes related to fatty acid synthesis; (**D**–**F**) Western blot analysis and quantification of FOXO1 and *p*-FOXO1/FOXO1 protein expression. * *p* < 0.05; ** *p* < 0.01.

RT-qPCR was further used to detect the expression of genes related to lipid metabolism. The results revealed that following *Sirt7* inhibition, the mRNA expression of *FOXO1*, a key transcription factor involved in milk fat synthesis, was significantly decreased (*p* < 0.05), whereas the mRNA expression of *SREBP1*, another key transcription factor regulating lipogenic gene expression, was highly significantly increased (*p* < 0.01, Figure 2A). For lipid transport and lipolysis pathways, *CD36* mRNA expression was highly significantly upregulated (*p* < 0.01), whereas *FABP1* expression was highly significantly decreased (*p* < 0.01, Figure 2B). In terms of fatty acid synthesis, the transcript levels of *ACC* and *SCD1* were highly significantly upregulated (*p* < 0.01), and *EVOLE6* expression was significantly upregulated (*p* < 0.05, Figure 2C). Consistent with the transcriptional changes, Western blot analysis verified that Sirt7 suppression highly significantly reduced the protein levels of total FOXO1 compared with the control group (*p* < 0.05, Figure 2D–F).

### 3.3. Effects of Sirt7 Inhibition on Triglycerides and Lipid Droplets

**Figure 3:**
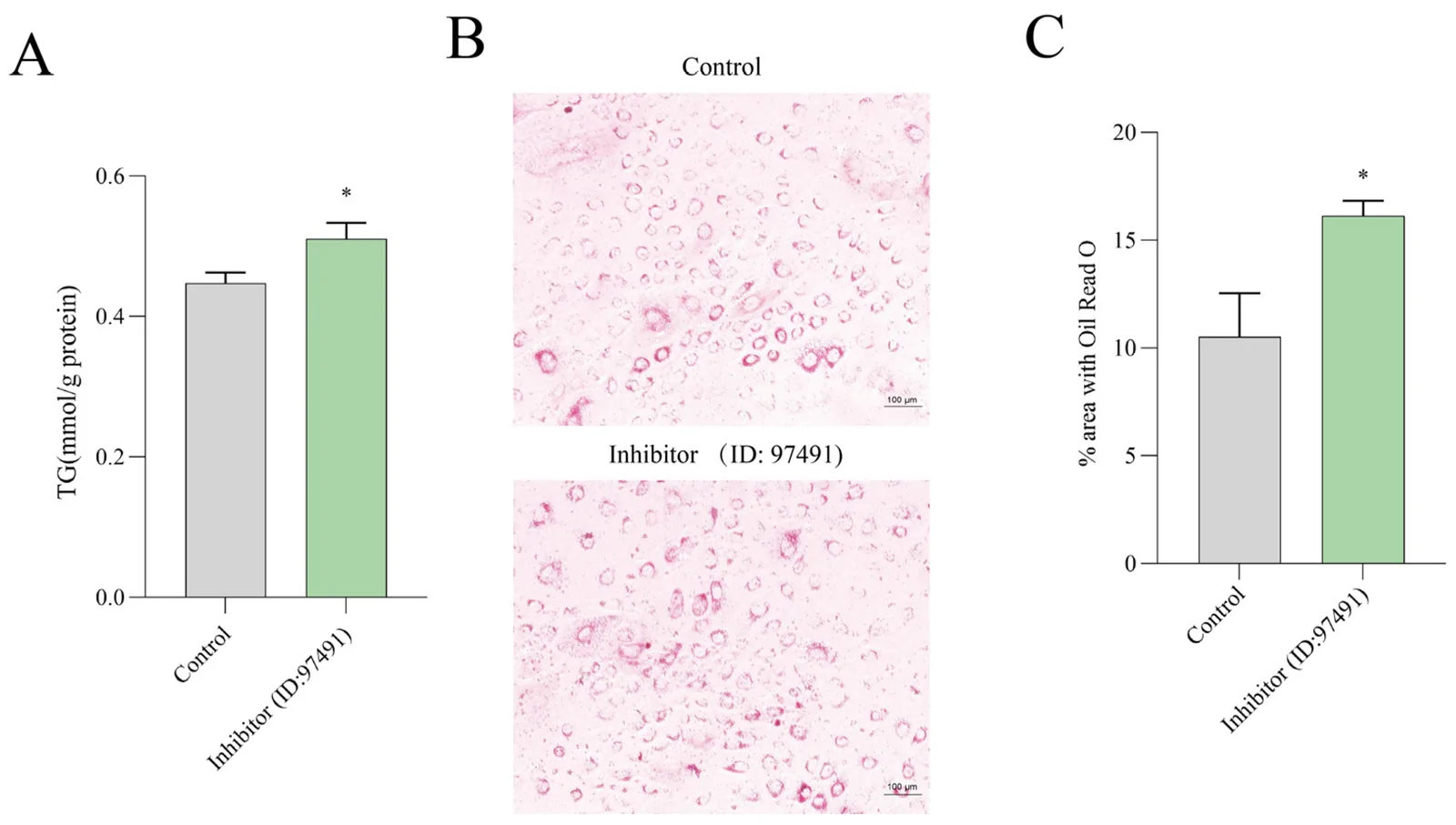
Sirt7 Inhibition Promotes Intracellular Lipid Accumulation in GMECs. (**A**) Relative intracellular triglyceride content after Sirt7 inhibition. (**B**,**C**) Oil red O staining and quantification of lipid droplets after Sirt7 inhibition. * *p* < 0.05.

To elucidate the role of Sirt7 in lipid deposition within goat mammary epithelial cells (GMECs), intracellular triglyceride (TG) content was measured following Sirt7 inhibition. The results demonstrated that TG content was significantly increased (*p* < 0.05, Figure 3A). Furthermore, Oil Red O staining revealed that, compared with the control group (0 μmol/L), treatment with the Sirt7 inhibitor (ID: 97491) resulted in increased intracellular lipid droplets and pronounced aggregation (*p* < 0.05, Figure 3B,C).

### 3.4. Effects of Sirt7 Inhibition on Fatty Acid Synthesis in GMECs

As shown in Table 2, inhibition of *Sirt7* significantly altered the contents of multiple fatty acids in GMECs compared with the control group. Among saturated fatty acids (SFA), the content of C11:0 was significantly decreased (*p* < 0.05), whereas the contents of C16:0, C15:0, C20:0 and C24:0 were significantly increased (*p* < 0.05). For monounsaturated fatty acids (MUFAs), the contents of C16:1 and C18:1n9c were significantly elevated (*p* < 0.05). In polyunsaturated fatty acids (PUFAs), the content of C20:5n3 was significantly reduced (*p* < 0.05). No significant differences were observed in the contents of other fatty acids between the treatment group and the control group.

## 4. Discussion

Inhibitor (ID: 97491) is a newly identified Sirt7 inhibitor that reduces Sirt7 activity [16]. Kim et al. [16] demonstrated that treatment with 5 and 10 μmol/L of the Sirt7 inhibitor (ID: 97491) for 72 h reduced cell proliferation by more than 50% in uterine sarcoma cells, while no significant effect on viability was observed in human embryonic kidney cells. However, in the present study, inhibitor (ID: 97491) at concentrations up to 15 μmol/L did not significantly affect the viability of GMECs. These results indicate that the cytotoxicity and cellular sensitivity to Sirt7 inhibitor (ID: 97491) may differ substantially across cell types.

FOXO1, as a key negative regulator of lipid metabolism, is involved in lipid synthesis in human [19], mice [20], and bovine tissues [21], and its expression affects TG synthesis and accumulation [22,23]. The results of the present study showed that *Sirt7* inhibition led to decreased *FOXO1* expression, which in turn promoted TG accumulation and lipid droplet formation. This finding is further supported by He et al. [24], who demonstrated that *FOXO1* knockdown significantly promoted TG synthesis and lipid droplet formation in GMECs, whereas *FOXO1* overexpression inhibited TG accumulation and reduced the number of lipid droplets. Additionally, a study on miR-145 in bovine mammary epithelial cells (BMECs) revealed that inhibition of its target gene *FOXO1* relieves the transcriptional repression of *SREBP1*, thereby promoting fatty acid synthesis and TG accumulation [25]. Collectively, these studies indicate that *FOXO1* acts as a key negative regulator of lipid accumulation in ruminant mammary epithelial cells, and its downregulation represents an important molecular mechanism promoting TG accumulation. Phosphorylation is the most common covalent post-translational modification of FOXO1. In MDA-MB-231 breast cancer cells, *Sirt7* knockdown enhances Akt phosphorylation and increases FOXO1 phosphorylation through the FKBP51 axis [26]. However, in the present study, *Sirt7* knockdown resulted in a marked reduction in total FOXO1 protein abundance, whereas the *p*-FOXO1/FOXO1 protein ratio remained largely unaffected. This discrepancy may be attributable to cell type-specific regulatory mechanisms, as well as the involvement of distinct upstream kinases or signaling cascades.

SREBP1 is a core transcription factor regulating milk fat synthesis, mediating the transcriptional expression of genes involved in cholesterol, fatty acid, and triglyceride synthesis [27]. The two isoforms of SREBP1 play distinct roles in lipid metabolism: *SREBP1a* primarily activates genes required for cholesterol and fatty acid synthesis (e.g., *ELOVL* and *SCD*), whereas *SREBP1c* regulates over 30 functional genes involved in fatty acid synthesis, transport, and triglyceride deposition, including *ACC*, *FASN*, and *SCD* [28]. Sirt7 negatively regulates the transcriptional activity of *SREBP1* through deacetylation; consequently, Sirt7 inhibition relieves this suppression and upregulates the expression of key lipogenic genes, including *SCD1* [29]. Activated SREBP1 initiates the expression of downstream lipogenic enzymes, thereby coordinately regulating fatty acid chain length and saturation. Specifically, ACC and FASN, as the rate-limiting enzymes of de novo fatty acid synthesis, mainly catalyze the production of medium-chain saturated fatty acids, such as C16:0 (palmitic acid) [30]. ELOVL6 further elongates C16:0 to long-chain saturated fatty acids, including C18:0 and C24:0 [31]. SCD1 then introduces double bonds into saturated fatty acyl chains via desaturation, catalyzing the generation of palmitoleic acid (C16:1) and oleic acid (C18:1n9c) [32]. The results of the present study are consistent with the above mechanisms: inhibition of *Sirt7* enhanced SREBP1 activity and consequently promoted triglyceride accumulation in GMECs. Downregulation of *Sirt7* activates the SREBP1-mediated lipogenic transcriptional network, leading to the coordinated upregulation of key genes involved in de novo synthesis, chain elongation, and desaturation. This, in turn, alters fatty acid chain length distribution and saturation, confirming that *Sirt7* negatively regulates milk fat synthesis and fatty acid composition remodeling in dairy goat mammary epithelial cells. In addition, PPARγ is also involved in the regulation of genes related to adipogenesis and lipid metabolism [33]. Studies have shown that *Sirt7*, as an upstream regulator of *PPARγ*, participates directly in the regulation of lipid synthesis by deacetylating *PPARγ2* at the K382 residue, thereby modulating the expression of lipogenesis-related genes in adipocytes and subsequently affecting adipogenesis and lipid metabolism [34]. However, in the present study, PPARγ expression in GMECs was not significantly affected following *Sirt7* inhibition. It is speculated that this discrepancy may be attributed to the fact that Sirt7 primarily regulates *PPARγ* at the level of post-translational deacetylation modification, rather than directly influencing its gene transcription.

In the present study, the expression levels of multiple lipid metabolism-related genes were observed to be significantly correlated with Sirt7 activity. *CD36*, *ACC*, *SCD1*, and *ELOVL6* are key functional genes in the fatty acid metabolic pathway, mediating fatty acid transmembrane transport, de novo lipogenesis, monounsaturated fatty acid synthesis, and long-chain fatty acid elongation, respectively, thereby co-regulating the synthesis process and fatty acid composition of goat milk fat [35,36,37,38]. The results showed that *Sirt7* inhibition significantly promoted the expression of these lipogenesis-related genes in GMECs. Concurrently, the expression of *FABP1*, involved in intracellular fatty acid transport, which mediates triglyceride hydrolysis, was significantly downregulated, indicating suppression of the lipolysis process [39,40]. Consequently, *Sirt7* inhibition simultaneously activates the lipogenic pathway and inhibits the lipolytic process, disrupting the homeostatic balance of lipid metabolism in GMECs and ultimately promoting the accumulation of intracellular triglycerides and lipid droplets.

Fatty acids are primarily classified into saturated fatty acids (SFAs), monounsaturated fatty acids (MUFAs), and polyunsaturated fatty acids (PUFAs) [41]. In the present study, inhibition of *Sirt7* resulted in substantial accumulation of medium- and long-chain fatty acids (including C14:0, C16:0, C20:0, C21:0, and C24:0) within GMECs. These findings are consistent with a previous study reporting significantly increased medium- and long-chain fatty acid levels in *Sirt7* knockout mice [42]. These alterations in fatty acid composition can modify the physicochemical properties and nutritional value of milk fat. Specifically, increased C16:0 (palmitic acid) enhances milk fat hardness and butter firmness, whereas increased C18:1n9c (oleic acid) lowers the melting point and improves the spreadability and texture of products such as butter and ice cream. The synergistic interaction between these two fatty acids determines the final processing characteristics of milk fat [43,44]. Meanwhile, the concurrent increase in both saturated fatty acids (SFAs) and monounsaturated fatty acids (MUFAs) reflects a physiological regulatory mechanism of the mammary gland, which serves to maintain milk fat fluidity and prevent excessive hardening [45]. From a functional perspective, the odd-chain fatty acid C15:0 serves as a characteristic biomarker of milk fat intake [46] and is associated with reduced risks of type 2 diabetes and metabolic syndrome [47]. Meanwhile, MUFAs such as C16:1 and C18:1c exert cardioprotective effects [48]. Although the decrease in the anti-inflammatory C20:5n3 content may partially compromise the anti-inflammatory nutritional value of milk fat, the overall fatty acid profile alterations induced by *Sirt7* inhibition are generally favorable for improving the nutritional quality and functional properties of milk [49]. Therefore, based on the results of the present study and previous findings, modulating *Sirt7* activity holds broad application prospects in dairy production. On one hand, targeted inhibition of *Sirt7* can activate the *SREBP1*-mediated lipogenic transcriptional network, thereby promoting triglyceride accumulation and the synthesis of medium-and long-chain fatty acids. This provides a novel molecular target for breeding strategies aimed at improving milk fat yield. On the other hand, the significant enrichment of beneficial fatty acids (C15:0, C16:1, and C18:1c) following *Sirt7* inhibition suggests that nutritional regulation or gene editing approaches could be employed to directionally optimize the fatty acid profile of goat milk. This would enable the customized production of functional dairy products enriched with bioactive fatty acids, thereby meeting consumer demands for cardiovascular protection and metabolic health benefits.

This study provides preliminary evidence, through inhibition, for a potential negative regulatory role of SIRT7 in milk fat synthesis in GMECs. However, the loss-of-function validation of SIRT7 was conducted solely using a chemical inhibitor, and bidirectional genetic evidence via CRISPR/Cas9-mediated gene editing or rescue experiments is lacking; therefore, the underlying mechanisms remain to be fully elucidated. Notably, Sirt7 inhibitor (ID: 97491) functions by suppressing the deacetylase activity of SIRT7, and the observed downregulation of SIRT7 transcript levels following treatment likely reflects a secondary response. As an NAD^+^-dependent deacetylase, SIRT7 has known substrates including histone H3K18 and non-histone proteins such as p53 and FOXO1. Based on the present findings, SIRT7 may regulate SREBP1 through two non-mutually exclusive mechanisms: either by directly deacetylating SREBP1 itself or its upstream transcriptional regulators or by modulating H3K18 acetylation levels in its promoter region. Future studies should combine SIRT7-knockout models with rescue experiments, ChIP-qPCR, and acetylation site-directed mutagenesis analysis for functional validation, and employ ChIP-seq to refine the regulatory network. Collectively, this study provides new molecular targets and a theoretical basis for the genetic improvement in milk quality traits in goats.

## 5. Conclusions

In this study, treatment of GMECs with the SIRT7 inhibitor (ID: 97491) resulted in downregulation of SIRT7 expression, accompanied by altered expression of SREBP1 and its downstream genes, a significant increase in intracellular triglyceride content, enhanced perinuclear lipid droplet aggregation, and systematic remodeling of the fatty acid profile. These results suggest that SIRT7 may be involved in the negative regulation of milk fat synthesis in GMECs, and that its inhibition is associated with triglyceride accumulation and fatty acid remodeling, potentially through activation of the SREBP1-mediated lipogenic transcriptional network. Collectively, this study provides preliminary evidence for a potential regulatory role of SIRT7 in caprine mammary lipid metabolism and lays a foundation for future mechanistic studies, as well as for the development of molecular targets aimed at improving milk fat yield and customizing functional dairy products.

## Figures and Tables

**Table 1:** Primer sequence information.

Gene	Gene ID	Primer Sequence (5′-3′)	Product Length (bp)
*Sirt7*	XM_018065444.1	F: GATTACCGGGGCCCTAATGG R: TCGCAGTTCTGAGACACCAC	158
*FOXO1*	MT577647.1	F: TGTCCTACGCCGACCTCATCAC R: CGGACACCTTTCTGAGTCAACCAC	96
*PPARγ*	XM_005681211.1	F: CCTTCACCACCGTTGACTTCT R: GATACAGGCTCCACTTTGATTGC	56
*SREBP1*	NM_001285755.1	F: AAGTGGTGGGCCTCTCTGA R: GCAGGGGTTTCTCGGACT	127
*ACC*	DQ370054.1	F: GGAGACAAACAGGGACCATT R: ATCAGGGACTGCCGAAAC	146
*FASN*	NM_001285629.1	F: TGTGCAACTGTGCCCTAG R: GTCCTCTGAGCAGCGTGT	111
*ELOVL6*	NM_001314257.1	F: TCTGGGCTTATGCATTTGTG R: CCAGCAACCATGTCCTTGTA	154
*SCD1*	KT315581.1	F: CCATCGCCTGTGGAGTCAC R: GTCGGATAAATCTAGCGTAGCA	256
*FABP1*	NM_001287558.1	F: CAAATACCAAGTCCAGACCCAG R: GCACGATTTCCGACACCC	113
*CD36*	NM_001285578.1	F: CCATCTTTGAACCCTCGC R: GGATCCGTATAGCCCCAC	196
*ATGL*	NM_001285739.1	F: GGTGCCAATATCATCGAGGT R: CACACCCGTGGCAGTCAG	133
*DGAT1*	MT221183.1	F: CCACTGGGACCTGAGGTGTC R: GCATCACCACACACCAATTCA	151
*HSL*	XM_062948346.1	F: AGGGTCATTGCCGACTTCC R: GTCTCGTTGCGTTTGTAGTGC	161
*UXT*	XM_005700842.2	F: CAGCTGGCCAAATACCTTCAA R: GTGTCTGGGACCACTGTGTCAA	125
*RPS9*	XM_005709411.1	F: CCTCGACCAAGAGCTGAAG R: CCTCCAGACCTCACGTTTGTTC	181
*MRPL39*	NM017446	F: AGGTTCTCTTTTGTTGGCATCC R: TTGGTCAGAGCCCCAGAAGT	101

Full gene names: Gene full names are as follows, sirtuin 7 (*Sirt7*), Forkhead box protein O1 (*FOXO1*), Peroxisome Proliferator-Activated Receptor γ (*PPARγ*), Sterol regulatory element-binding protein 1 (*SREBP1*), Acetyl-CoA carboxylase alpha (*ACC*), Fatty acid synthase (*FASN*), Elongation of very long chain fatty acids protein 6 (*ELOVL6*), stearoyl-CoA desaturase 1 (*SCD1*), Fatty acid-binding protein 1 (*FABP1*), Cluster of differentiation 36 (*CD36*), Adipose triglyceride lipase (*ATGL*), Diacylglycerol O-acyltransferase 1 (*DGAT1*), Hormone-sensitive lipase (*HSL*), Ubiquitously expressed transcript protein (*UXT*), Ribosomal protein S9 (*RPS9*), Mitochondrial ribosomal protein L39 (*MRPL39*).

**Table 2:** Effect of inhibitor 9749 on the fatty acid composition of GMECs (%).

Fatty Acids	Control Group	Treatment Group	*p*-Value
medium chain fatty acids			
C10:0	0.0061 ± 0.0013	0.0056 ± 0.0019	0.719
C12:0	0.3278 ± 0.048	0.2674 ± 0.046	0.19
SFAs			
C11:0	0.0378 ± 0.0024 ^a^	0.0202 ± 0.0004 ^b^	0.020
C13:0	0.0378 ± 0.0047	0.0271 ± 0.0065	0.083
C14:0	0.9145 ± 0.0739	1.1187 ± 0.1738	0.134
C15:0	0.1659 ± 0.0172 ^b^	0.226 ± 0.0261 ^a^	0.028
C16:0	32.6097 ± 3.4503 ^b^	39.0797 ± 1.9834 ^a^	0.048
C17:0	0.3886 ± 0.0139	0.3118 ± 0.0497	0.061
C18:0	16.5522 ± 1.2673	15.1387 ± 2.4828	0.429
C20:0	0.5501 ± 0.017 ^b^	0.637 ± 0.0424 ^a^	0.030
C21:0	0.0206 ± 0.0038	0.0186 ± 0.0037	0.545
C22:0	0.7433 ± 0.0889	0.7013 ± 0.1069	0.628
C23:0	0.0646 ± 0.013	0.0546 ± 0.0121	0.388
C24:0	0.8112 ± 0.0372 ^b^	0.9271 ± 0.0282 ^a^	0.013
MUFAs			
C16:1	0.8753 ± 0.1186 ^b^	1.2382 ± 0.1403 ^a^	0.027
C17:1	0.5008 ± 0.1353	0.3669 ± 0.0386	0.175
C18:1n9t	0.6674 ± 0.2143	0.4598 ± 0.0576	0.180
C18:1n9c	20.5825 ± 2.6894 ^b^	25.7349 ± 1.6552 ^a^	0.048
C20:1	0.0216 ± 0.0055	0.0184 ± 0.0042	0.463
C22:1n9	7.0592 ± 1.2173	6.3381 ± 0.7423	0.430
C24:1	0.8411± 0.0899	0.7921 ± 0.0496	0.455
PUFAs			
C18:2n6c	4.6204 ± 0.7169	4.7656 ± 0.3919	0.774
C18:3n3	0.086 ± 0.0102	0.1105 ± 0.0255	0.198
C20:3n6	0.6025 ± 0.0679	0.8933 ± 0.2099	0.085
C20:4n6	2.971 ± 0.2937	2.4488 ± 0.449	0.167
C22:2	0.1857 ± 0.0214	0.2106 ± 0.0319	0.232
C20:5n3	0.5465 ± 0.0764 ^b^	0.3876 ± 0.0542 ^a^	0.042
C22:6n3	1.5644 ± 0.1719	1.6644 ± 0.2052	0.553

Note: Different lowercase letters indicate significant differences between groups (*p* < 0.05).

## Data Availability

The data presented in this study are available from the corresponding author upon request.
